# Dynamic interaction of multiple shear bands

**DOI:** 10.1038/s41598-018-34322-w

**Published:** 2018-10-30

**Authors:** Diana Giarola, Domenico Capuani, Davide Bigoni

**Affiliations:** 1 University of Trento, DICAM, Trento, I-38123 Italy; 2 University of Ferrara, DA, Ferrara, I-44121 Italy

## Abstract

A mechanical model for waves impinging different configurations of multiple shear bands already formed in a ductile material, allows to analyze the ways in which dynamic interactions promote failure. It is shown that the presence of more than one shear band may lead to resonance and correspondent growth of a shear band or, conversely, to its annihilation. At the same time, multiple scattering may bring about focusing or, conversely, shielding from waves. The proposed mechanical modelling, represents the only way to analyze the fine micromechanisms governing material collapse, and discloses the complex interplay between dynamics and shear band growth or arrest.

## Introduction

Nucleation and growth of shear bands and their interactions in ductile materials are concurrent causes of failure^1^, a complex process which is strongly affected by dynamics and far from being completely understood.

Interaction between shear bands has been documented so far only for quasi-static deformation processes^2–4^, where it has been shown that different shear band geometries emerge as related to load conditions and material properties of the samples and that parallel^5^, aligned, and converging shear bands^6^ are frequently observed. In dynamics, results are restricted to high strain-rate loading, where numerical simulations^7–9^ and experiments involving impact on prenotched plates^10–12^ have been presented. In this context, experiments on metallic glass^13^ show the development of a complex texture of multiple shear bands, with complex interactions.

Direct experimental investigation on the fine development of shear bands in a material and their effect on the stress field during time-harmonic vibrations remains practically impossible, so that mechanical modelling represents the only possibility to shed light on a complex phenomenon, whose comprehension is a key point for the engineering of materials with enhanced mechanical properties.

A plane-strain model of multiple shear bands, arranged in different configurations, commonly observed in materials and involving two or four shear bands, is introduced in the present article, to investigate the dynamical interplay between shear bands and their possible progression or stagnation. Reference is made to low to medium carbon steels, stressed until near the verge of a plastic instability and subject to incoming harmonic waves of small amplitude. The material is modelled following the J_2_-deformation theory of plasticity^14^, the shear bands are idealized as discontinuity surfaces^15^, and their dynamic interaction is described through an *ad hoc* integral equation formulation, presented together with the relevant numerical method.

Results of simulations unveil the complex interactions developing between multiple shear bands during wave propagation, leading in some cases to resonance (which promotes shear band growth and coalescence), but in other cases to annihilation (which produces shear band arrest). Furthermore, different geometries can lead to opposite effects, of focusing or shielding from waves, so that in the former case nucleation of a new shear band is promoted, while in the latter the material remains ‘untouched’ by the wave field.

## Wave impinging different configurations of shear bands

**Figure 1:**
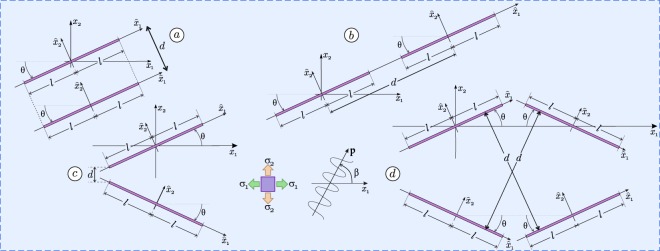
Waves (inclined at the angle *β*) impinging different configurations of shear bands (of equal length 2*l*) in a prestressed metal material: (**a**) parallel, (**b**) aligned, (**c**) converging, and (**d**) involving 4 shear bands.

The incremental response of a metallic material subject to a state of initial stress (so-called ‘prestress’) can be modelled through the J_2_-deformation theory of plasticity^14–16^. According to this material model, shear bands are inclined (with respect to the principal stress axes) at the angles

1 $$\theta =\pm \,{\rm{arccot}}\sqrt{\frac{1+2\,{\rm{sign}}(k)\sqrt{\xi \mathrm{(1}-\xi )}}{1-2\xi }},$$

where *k* and *ξ* = *μ*/μ_*_ are parameters ruling the state of deviatoric prestress and the incompressible material anisotropy, respectively (*μ* and μ_*_ are shear moduli parallel to, and inclined with 45° to, the principle stress axis). Three commonly observed^5,6^ configurations of shear bands embedded in the material are analyzed, namely, (a) parallel, (b) aligned and (c) converging geometries, arranged as shown in Fig. 1.

The modelling of *one* shear band was previously proposed^15^, and the dynamic formulation^17^ is extended here to deal with interactions between several shear bands (see the theoretical formulation presented in the supporting electronic material), providing the governing system of boundary integral equations and the relevant collocation method for the numerical solution, both properly capturing the stress singularity at the shear band tips (the treatment is deferred to the section *Boundary integral equation and numerical solution*).

Results reported in the following are limited for simplicity to the hardening exponent *N* = 0.4, representative of a medium carbon steel, and to a level of prestress close to the elliptic boundary (*k* =  0.87 and *ξ* = 0.26, parameters corresponding to the inclination *θ* ≈ ±26°), so that some shear bands are expected to be already formed. Time-harmonic incident shear waves of circular frequency Ω selected as characterized by an incremental displacement field **v**^*inc*^(**x**), with amplitude *A* and phase velocity *c*, propagation direction **p** and direction of motion **d**^18^

2 $${\bf{v}}=A{\bf{d}}{e}^{i\frac{{\rm{\Omega }}}{c}({\bf{x}}\cdot {\bf{p}}-ct)}.$$

### Parallel shear bands

**Figure 2:**
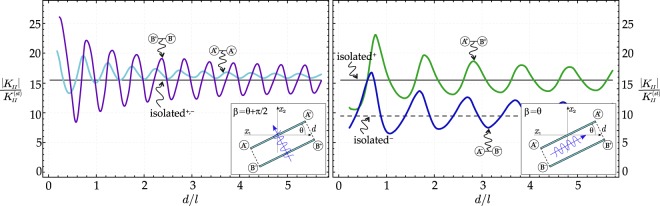
Resonance, as induced by two parallel shear bands (much more pronounced than in the case of an isolated shear band, also reported in the figure), is revealed by the peaks of the dimensionless stress intensity factor, reported as a function of the distance *d* between the shear bands for a wavenumber Ω*l*/*c*_1_ = 1: (**a**) direction of the wave propagation orthogonal to the shear bands *β* = *θ* + *π*/2, (**b**) direction of the wave propagation parallel to the shear bands *β* = *θ*. The SIF of one isolated shear band is also reported. The right and left tips are labelled with + and − respectively.

In the case of two equal and parallel shear bands (Fig. 1(a)), two directions of propagation for the impinging waves with wavenumber Ω*l*/*c*_1_ = 1 (*c*_1_ is the propagation velocity in the direction of *x*_1_-axis) are considered: one aligned orthogonal (*β* = *θ* + *π*/2) and the other parallel to the shear bands (*β* = *θ*). The ratio between the static and dynamic Mode II Stress Intensity Factor^17^ (briefly denoted with ‘SIF’ in the following) at the tip of the shear band is reported in Fig. 2, as a function of the dimensionless distance *d*/*l* between the two shear bands. At the tips *A*^−^, *A*^+^ and *B*^−^, *B*^+^ the values of the SIF oscillate about the value pertinent to an isolated shear band. Note that the SIF assumes the same value

- at the tip pairs *A*^−^, *A*^+^ and *B*^−^, *B*^+^ for wave propagation orthogonal to the shear bands;
- at the tip pairs *A*^−^, *B*^−^ and *A*^+^, *B*^+^ for wave propagation parallel to the shear bands.

For both wave propagation directions, the SIFs grow when the distance between the two shear bands decreases. The peaks of the SIFs denote resonance, which is much more pronounced than in the case of an isolated shear band (reported in Fig. 2 for comparison^17^). Therefore, two shear bands provide an amplification to resonance, thus promoting shear band growth.

**Figure 3:**
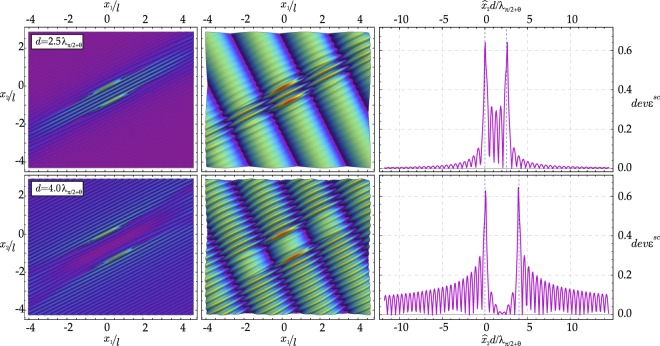
Examples of wave focusing (upper part) and shielding (lower part) generated by two parallel shear bands. Scattered (left) and total (centre) incremental deviatoric strain field is reported as produced by an incident shear wave travelling parallel to the shear bands (*β* = *θ*) with wave number Ω*l*/*c*_1_ = 1. The graphs on the right side are cross-sections of maps on the left side, cut at the centre of the shear bands.

For the shear band geometry (a), the (modulus of the) incremental deviatoric strain is plotted in Fig. 3, when a wave is travelling parallel to the shear band ensemble. The two cases reported in the figure differ only in the distance between the shear bands. In the upper and lower parts of Fig. 3, the distance is *d* = 2.5*λ*_*π*/2+*θ*_ and *d* = 4*λ*_*π*/2+*θ*_, respectively, with *λ*_*α*_ being the wavelength in the propagation direction singled out by angle *α*. The scattered field is reported on the left, while the total field in the centre. The graphs on the right side of Fig. 3 are cross-sections of the scattered deviatoric strain along ${\hat{x}}_{2}$-axis, cut at shear band centre.

Overall, Fig. 3 shows that the ratio between the shear band distance and the wavelength of the impinging wave may determine focusing (which promotes shear band nucleation, see the upper part of the figure) or shielding (which leaves the material inside the shear bands unstressed, see the lower part of the figure) of the mechanical disturbance in the region enclosed within the shear bands. Similar results for the SIF can be found in fracture mechanics, for the dynamics of parallel cracks. In particular, increasing values of the SIF at decreasing crack distance^19–22^ and focussing of the displacement field between the cracks^23^ have been found.

### Aligned shear bands

**Figure 4:**
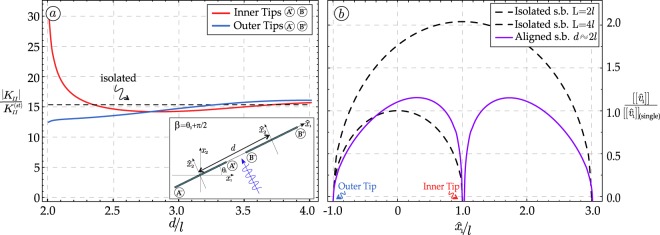
(**a**) Coalescence of two aligned shear bands is demonstrated by the dimensionless SIF reported as a function of the distance *d* for a wave travelling orthogonal to the shear bands and wavenumber Ω*l*/*c*_1_ = 1; (**b**) The strong difference between two aligned shear bands of length 2*l* close to each other, and one isolated shear band, either of length 4*l* or 2*l* (both reported as dashed lines) is visible from the profile of the displacement jump along the shear band surfaces.

Two aligned shear bands of equal length 2*l*, at a distance *d*, are analyzed (Fig. 1(b)) for an impinging wave with wavenumber Ω*l*/*c*_1_ = 1. The normalized SIF at the tips of the shear bands is evaluated as a function of the dimensionless distance *d*/*l*, for an incident wave with direction of propagation orthogonal to the shear bands (*β* = *θ* + *π*/2), Fig. 4(a). When the two shear bands are distant, the SIFs tend to the value pertinent to one isolated shear band, but the SIF at the inner tips (*A*^+^*B*^−^) strongly blows up, when the distance between the two shear bands tends to vanish. This effect promotes the *coalescence of the two shear bands*.

For a wave travelling orthogonal to two aligned shear bands, the profile of the displacement jump across the shear band surfaces is reported in Fig. 4(b), for *d* = 2*l*(1 + 1/100) ≈ 2*l*, together with the profile pertinent to an isolated shear band of length 2*l* and 4*l*. The profiles of the two aligned shear bands are non-symmetric and, in a sense, they seem to ‘attract each other’. However, the two profiles are very different, so that two shear bands are not equivalent to an isolated shear band with a length equal to the sum of the two lengths.

**Figure 5:**
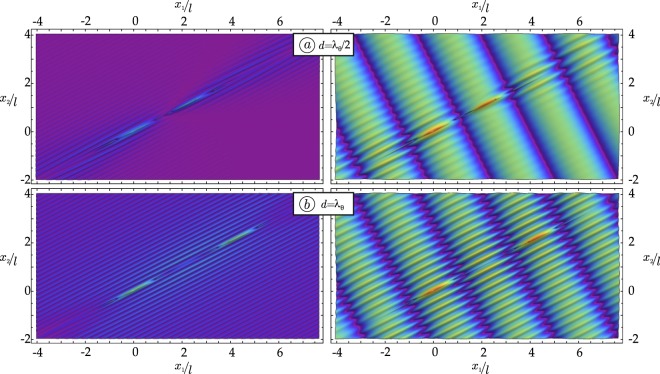
(**a**) Annihilation of the stress field is revealed by the modulus of the incremental deviatoric strain field produced by an incident shear wave travelling parallel to the shear bands (*β* = *θ*) with wave number Ω*l*/*c*_1_ = 1 and distance *d* = *λ*_*θ*_/2; (**b**) Amplification of the stress field is determined under the same condition as for (**a**), but assuming *d* = *λ*_*θ*_. Note that the maps on the left represent the scattered field, while the total fields are reported on the right.

The (modulus of the) total deviatoric strain field is reported for two aligned shear bands in Fig. 5(a,b), for a wave travelling parallel to them (*β* = *θ*), both for the case with *d* = *λ*_*θ*_/2 and for the case with *d* = *λ*_*θ*_. These figures show a sort of overall’ elimination or intensification, meaning that the scattered field is everywhere annihilated in case (a) or amplified in case (b). Therefore, a system of two shear bands produces a mechanical disturbance which may or may not (depending on the ratio *d*/*λ*) propagate in a material far beyond the location of the shear bands.

### Converging shear bands

**Figure 6:**
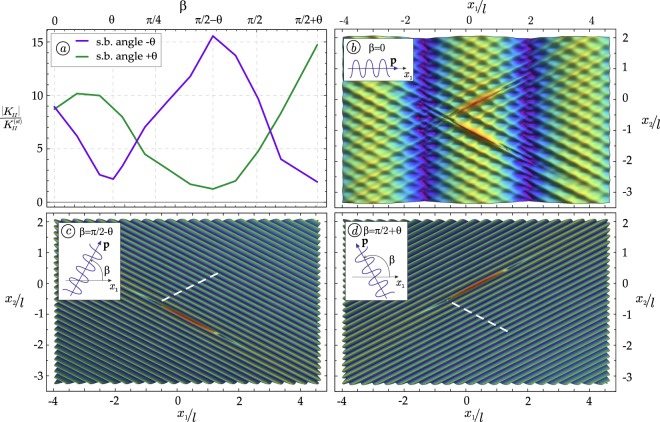
(**a**) Loading and simultaneous unloading of two converging shear band tips is revealed by the dimensionless Mode II SIF, reported at the closest tips of both shear bands, as function of the propagation direction *β* of the impinging wave; (**b**) A fine texture of secondary planar waves is evidenced by the modulus of the deviatoric incremental strain field produced by an impinging wave propagating horizontally *β* = 0; (**c**) Annihilation of one shear band and reinforcement of the other is produced in the same conditions as for part (**b**), but assuming *β* = *π*/2 − *θ*; (**d**) A switch is produced from the annihilated to the reinforced shear band with respect to part (**c**), assuming now *β* = *π*/2 + *θ*.

Two shear bands, disposed in a conical geometry, inclined at ±*θ* and located at a minimal distance *d* = *l*/10 (Fig. 1(c)), are analyzed in Fig. 6, for Ω*l*/*c*_1_ = 1. The dimensionless SIF is reported in Fig. 6(a), as a function of the propagation direction *β* of the impinging wave, and shows that when the wave travels orthogonal to one of the two shear bands (*β* = *π*/2 − *θ* or *β* = *π*/2 + *θ*), the relevant shear band tip is loaded with a maximum value of the shear stress, while the other shear band tip results unloaded. This effect, which corresponds to the annihilation of a shear band, becomes clearly visible in parts (c) and (d) of the figure, where one shear band (marked with a dashed white line) ‘disappears’, while at the same time the other is ‘reinforced’. When the impinging wave is horizontal, the two shear bands behave in the same way and produce a fine texture of ‘secondary’ planar waves inclined at the critical directions for shear band formation, as is shown in Fig. 6(b).

### Four shear bands

**Figure 7:**
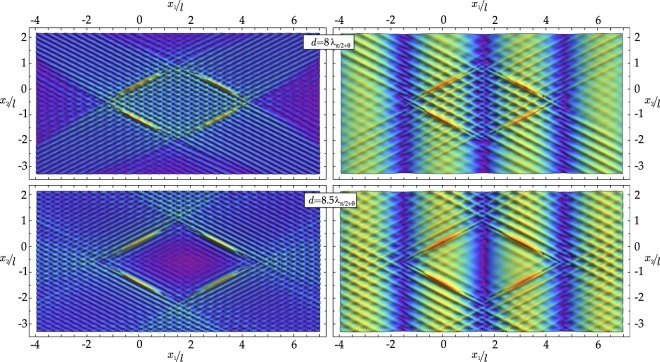
Examples of creation of an ‘island’ of focusing (upper part) and shielding (lower part) produced by a system of four shear bands subjected to horizontally (*β* = 0) impinging waves. Scattered (left) and total (right) incremental deviatoric strain field are reported, for wave number Ω*l*/*c*_1_ = 1: in the upper part, a characteristic shear band distance *d* = 8*λ*_*π*/2+*θ*_ is selected, while *d* = 8.5*λ*_*π*/2+*θ*_ is selected for the lower part.

A system of four shear bands are considered as sketched in Fig. 1(d), impinged by a horizontally propagating wave with Ω*l*/*c*_1_ = 1. Results in terms of maps of the (modulus of the) deviatoric strain are reported in Fig. 7 for the scattered (left) and total (right) fields at a distance *d* = 8*λ*_*π*/2+*θ*_ (upper part) and *d* = 8.5*λ*_*π*/2+*θ*_ (lower part). The case reported in the upper part of the figure provides an example of focusing of the signal, while shielding is evidenced in the case reported in the lower part. In the upper case, the stress intensifies, while in the lower case an ‘island of stress relief’ is created.

## Boundary integral equation and numerical solution

The boundary integral equation governing the dynamics of a shear band^17^ is (in terms of Hadamard finite part integral)

3 $${\hat{t}}_{21}^{(inc)}({\bf{y}})={n}_{l}{s}_{m}{{\mathbb{K}}}_{lmkg}{\int }_{-l}^{l}{\dot{t}}_{ij,k}^{g}\,({\hat{x}}_{1},{\bf{y}}){n}_{i}{s}_{j}\,[\kern-2pt[ {\hat{v}}_{1}]\kern-2pt] \,d{\hat{x}}_{1},$$

where ${\hat{t}}_{21}^{(inc)}$ is the nominal incremental shear impinging the surface of the shear band, ${\mathbb{K}}$ is the constitutive forth-order tensor (relating the gradient of incremental displacement to the increment of nominal stress), **n** and **s** are the normal and tangential unit vector, respectively, to the shear band surface, $[\kern-2pt[ {\hat{v}}_{1}]\kern-2pt]$ is the unknown tangential displacement jump across the shear band faces and ${\dot{t}}_{ij,k}^{g}$ is the stress gradient of the incremental Green function^24^.

In the case of multiple shear bands, labelled *A*, *B*, ..., *Z*, in order to account for the reciprocal interaction, the equation (3) has to be generalized to the following system of integral equations

4 $$\begin{array}{rcl}{\hat{t}}_{21}^{(inc)}({{\bf{y}}}^{A}) & = & {n}_{l}^{A}{s}_{m}^{A}{{\mathbb{K}}}_{lmkg}({\int }_{-l}^{l}{\dot{t}}_{ij,k}^{g}({\hat{x}}_{1}^{A},{{\bf{y}}}^{A}){n}_{i}^{A}{s}_{j}^{A}[\kern-2pt[ {\hat{v}}_{1}^{A}]\kern-2pt] d{\hat{x}}_{1}^{A}\\  &  & +{\int }_{-l}^{l}{\dot{t}}_{ij,k}^{g}({\hat{x}}_{1}^{B},{{\bf{y}}}^{A}){n}_{i}^{B}{s}_{j}^{B}[\kern-2pt[ {\hat{v}}_{1}^{B}]\kern-2pt] \,d{\hat{x}}_{1}^{B}+\ldots \\  &  & +{\int }_{-l}^{l}{\dot{t}}_{ij,k}^{g}({\hat{x}}_{1}^{Z},{{\bf{y}}}^{A}){n}_{i}^{Z}{s}_{j}^{Z}\,[\kern-2pt[ {\hat{v}}_{1}^{Z}]\kern-2pt] d{\hat{x}}_{1}^{Z}),\\ {\hat{t}}_{21}^{(inc)}({{\bf{y}}}^{B}) & = & {n}_{l}^{B}{s}_{m}^{B}{{\mathbb{K}}}_{lmkg}({\int }_{-l}^{l}{\dot{t}}_{ij,k}^{g}({\hat{x}}_{1}^{A},{{\bf{y}}}^{B}){n}_{i}^{A}{s}_{j}^{A}[\kern-2pt[ {\hat{v}}_{1}^{A}]\kern-2pt] d{\hat{x}}_{1}^{A}\\  &  & +{\int }_{-l}^{l}{\dot{t}}_{ij,k}^{g}({\hat{x}}_{1}^{B},{{\bf{y}}}^{B}){n}_{i}^{B}{s}_{j}^{B}[\kern-2pt[ {\hat{v}}_{1}^{B}]\kern-2pt] d{\hat{x}}_{1}^{B}+\ldots \\  &  & +{\int }_{-l}^{l}{\dot{t}}_{ij,k}^{g}({\hat{x}}_{1}^{Z},{{\bf{y}}}^{B}){n}_{i}^{Z}{s}_{j}^{Z}[\kern-2pt[ {\hat{v}}_{1}^{Z}]\kern-2pt] d{\hat{x}}_{1}^{Z}),\\ \ldots  &  & \\ {\hat{t}}_{21}^{(inc)}({{\bf{y}}}^{B}) & = & {n}_{l}^{Z}{s}_{m}^{Z}{{\mathbb{K}}}_{lmkg}({\int }_{-l}^{l}{\dot{t}}_{ij,k}^{g}({\hat{x}}_{1}^{A},{{\bf{y}}}^{Z}){n}_{i}^{A}{s}_{j}^{A}[\kern-2pt[ {\hat{v}}_{1}^{A}]\kern-2pt] d{\hat{x}}_{1}^{A}\\  &  & +{\int }_{-l}^{l}{\dot{t}}_{ij,k}^{g}({\hat{x}}_{1}^{B},{{\bf{y}}}^{Z}){n}_{i}^{B}{s}_{j}^{B}[\kern-2pt[ {\hat{v}}_{1}^{B}]\kern-2pt] d{\hat{x}}_{1}^{B}+\ldots \\  &  & +{\int }_{-l}^{l}{\dot{t}}_{ij,k}^{g}({\hat{x}}_{1}^{Z},{{\bf{y}}}^{Z}){n}_{i}^{Z}{s}_{j}^{Z}[\kern-2pt[ {\hat{v}}_{1}^{Z}]\kern-2pt] d{\hat{x}}_{1}^{Z}),\end{array}$$

where the generic source point **y**^*Z*^ is located on the surface of the shear band *Z*.

For the numerical solution, each shear band has been subdivided into a number *Q* of elements. In order to describe the square-root singularity^25^, two different kinds of shape function have been adopted: quadratic function for the elements interior to the shear band and square-root function (the so called ‘quarter-point element’s’^26,27^) at the shear band tips.

Using a collocation method, the integral equation system (4), can be transformed into the following algebraic system

5 $$\{\begin{array}{c}{\hat{{\bf{t}}}}_{21}^{{(inc)}^{A}}\\ {\hat{{\bf{t}}}}_{21}^{{(inc)}^{B}}\\ \ldots \\ {\hat{{\bf{t}}}}_{21}^{{(inc)}^{Z}}\end{array}\}=[\begin{array}{cccc}{{\bf{C}}}_{AA} & {{\bf{C}}}_{AB} & \ldots  & {{\bf{C}}}_{AZ}\\ {{\bf{C}}}_{BA} & {{\bf{C}}}_{BB} & \ldots  & {{\bf{C}}}_{BZ}\\ \ldots  & \ldots  & \ldots  & \ldots \\ {{\bf{C}}}_{ZA} & {{\bf{C}}}_{ZB} & \ldots  & {{\bf{C}}}_{ZZ}\end{array}]\,\{\begin{array}{c}[[{\hat{{\bf{v}}}}^{A}]]\\ \,[[{\hat{{\bf{v}}}}^{B}]]\\ \ldots \\ \,[[{\hat{{\bf{v}}}}^{Z}]]\end{array}\},$$

where ${\hat{{\bf{t}}}}_{21}^{{(inc)}^{n}}$ is the known nodal value of the incident traction on the *n*-th shear band, $[[{\hat{{\bf{v}}}}^{n}]]$ is the unknown nodal value of the displacement jump across the *n*-th shear band, and **C** is the coefficient matrix with out-of-diagonal sub-matrices, collecting the contributions of the dynamic interaction between the shear bands. A validation of the collocation method, with reference to other numerical and analytical solutions, is reported in the supplementary material.

Finally, note that although the integral equation and numerical procedure are limited in the present study to two-dimensional continua, results can be extended to three dimensional deformation by using the 3D Green’s function^28^.

## Electronic supplementary material

Mathematical details on the mechanical model

## References

[1] Petryk H, Kursa M (2013). The energy criterion for deformation banding in ductile single crystals. J. Mech. Phys. Solids.

[2] Dolinski M, Merzer M, Rittel D (2015). Analytical formulation of a criterion for adiabatic shear failure. Int. J. Impact Eng..

[3] He J (2016). Local microstructure evolution at shear bands in metallic glasses with nanoscale phase separation. Scientific Reports.

[4] Hsieh TH, Kinloch AJ, Masania K, Taylor AC, Sprenger S (2010). The mechanisms and mechanics of the toughening of epoxy polymers modified with silica nanoparticles. Polymer.

[5] Li W, Gao Y, Bei H (2016). Instability Analysis and Free Volume Simulations of Shear Band Directions and Arrangements in Notched Metallic Glasses. Scientific Reports.

[6] Qu RT, Liu ZQ, Wang G, Zhang ZF (2015). Progressive shear band propagation in metallic glasses under compression. Acta Materialia.

[7] Bonnet-Lebouvier AS, Molinari A, Lipinski P (2002). Analysis of the dynamic propagation of adiabatic shear bands. Int. J. Solids Structures.

[8] Li S, Liu WK, Qian D, Guduru PR, Rosakis AJ (2001). Dynamic shear band propagation and micro- structure of adiabatic shear band. Comput. Methods Appl. Mech. Eng..

[9] Needleman A (1989). Dynamic Shear Band Development in Plane Strain. J. Appl. Mech..

[10] Guduru PR, Rosakis AJ, Ravichandran G (2001). Dynamic shear bands: an investigation using high speed optical and infrared diagnostic. Mech. Materials.

[11] Ma M, Vijayan K, hiltner A, Baer E (1989). Shear yielding modes of polycarbonate. J. Mat. Sci..

[12] Zhou M, Rosakis AJ, Ravichandran G (1996). Dynamically propagating shear bands in impact-loaded prenotched plates. II-Numerical simulations. J. Mech. Phys. Solids.

[13] Ruan HH, Zhang LC, Lu J (2011). A new constitutive model for shear banding instability in metallic glass. Int. J. Solids Structures.

[14] Bigoni, D. Nonlinear Solid Mechanics Bifurcation Theory and Material Instability. Cambridge University Press (2012).

[15] Bigoni D, Dal Corso F (2008). The unrestrainable growth of a shear band in a prestressed material. Proc. R. Soc. A.

[16] Hill R (1958). A general theory of uniqueness and stability in elastic-plastic solids. J. Mech. Phys. Solids.

[17] Giarola D, Capuani D, Bigoni D (2018). The dynamics of a shear band. J. Mech. Phys. Solids.

[18] Ogden R, Singh B (2011). Propagation of waves in an incompressible transversely isotropic elastic solid with initial stress: Biot revisited. J. Mech. Materials Struct..

[19] Dineva P, Gross D, Rangelov T (2008). Dynamic interaction of cracks in piezoelectric and anisotropic solids: a non-hypersingular BIEM approach. Theoret. Appl. Mech..

[20] Garcia-Sanchez F, Saez A, Dominguez J (2006). Two-dimensional time-harmonic BEM for cracked anisotropic solids. Engineering Analysis with Boundary Elements.

[21] Gross D, Zhang CH (1986). Diffraction of SH waves by a system of cracks: Solution by an integral equation method. Int. J. Solids Structures.

[22] Phan AV (2016). Dynamic stress intensity factor analysis of the interaction between multiple impact-loaded cracks in infinite domains. AIMS Mat. Sci..

[23] Rojas-Diaz R, Garcia-Sanchez F, Saez A (2009). Dynamic crack interactions in magnetoelectroelastic composite materials. Int. J. Frac..

[24] Bigoni D, Capuani D (2005). Time-harmonic Green’s function and boundary integral formulation for incremental nonlinear elasticity: dynamics of wave patterns and shear bands. J. Mech. Phys. Solids.

[25] Salvadori A, Gray LJ (2007). Analytical integrations and SIFs computation in 2D fracture mechanics. Comput. Meth. Appl. Mech. Engrg..

[26] Chirino F, Abascal R (1998). Dynamic and static analysis of cracks using the hypersingular formulation of the boundary element method. Int. J. Num. Meth. Engng..

[27] Salvadori A (2002). Analytical integrations in 2D BEM elasticity. Comput. Meth. Appl. Mech. Engrg..

[28] Argani L, Bigoni D, Capuani D, Movchan NV (2014). Cones of localized shear strain in incompressible elasticity with prestress: Green’s function and integral representations. Proc. R. Soc. A.

