# Supplementary material for: Dynamic interaction of multiple shear bands
Source: Sci Rep. 2018 Oct 30;8:16033. doi: 10.1038/s41598-018-34322-w (PMC6207654; doi:10.1038/s41598-018-34322-w)
Supplement: Supplementary file 1 — Mathematical details on the mechanical model [file 41598_2018_34322_MOESM1_ESM.pdf]

Scientific Report

Dynamic interaction of multiple shear bands

Supplementary material

Diana Giarola[1], Domenico Capuani[2], Davide Bigoni[1]
(1) DICAM, University of Trento, via Mesiano 77, I-38050 Trento, Italy
(2) DA, University of Ferrara, via Quartieri 8, I-44121 Ferrara, Italy
e-mail: diana.giarola@unitn.it, bigoni@ing.unitn.it, domenico.capuani@unife.it

1 Incremental constitutive equations and dynamic Green's functions

When plane strain conditions prevail, the constitutive equation of a hyperelastic incompressible material can be expressed as

$$\dot{t}_{ij} = \mathbb{K}_{ijkl} v_{l,k} + \dot{p}\delta_{ij}, \quad v_{i,i} = 0, \tag{1}$$

where indices range between 1 and 2, v_i is the incremental displacement, $\dot{t}_{ij}$ is the incremental unsymmetric Piola stress, $\dot{p}$ the incremental, in-plane mean stress and δ_{ij} the Kronecker delta. The fourth-order tensor $\mathbb{K}_{ijkl}$ of the instantaneous moduli, possesses the major symmetry $\mathbb{K}_{ijkl} = \mathbb{K}_{klij}$, and its non-null components are defined as

$$\begin{aligned}
&\mathbb{K}_{1111} = \mu_* - \frac{\sigma}{2} - p, && \mathbb{K}_{1122} = -\mu_*, && \mathbb{K}_{1112} = \mathbb{K}_{1121} = 0,\\
&\mathbb{K}_{2211} = -\mu_*, && \mathbb{K}_{2222} = \mu_* + \frac{\sigma}{2} - p, && \mathbb{K}_{2212} = \mathbb{K}_{2221} = 0,\\
&\mathbb{K}_{1212} = \mu + \frac{\sigma}{2}, && \mathbb{K}_{1221} = \mathbb{K}_{2112} = \mu - p, && \mathbb{K}_{2121} = \mu - \frac{\sigma}{2},
\end{aligned} \tag{2}$$

where μ and μ_* are two incremental shear moduli, respectively parallel and inclined at 45° with respect to the x_1-axis, and the prestress parameters σ and p are the in-plane deviatoric and mean stresses, functions of the principal Cauchy

stresses, respectively, defined as

$$\sigma = \sigma_1 - \sigma_2, \qquad p = \frac{\sigma_1 + \sigma_2}{2}. \tag{3}$$

The equations of incremental motion can be written as

$$\dot{t}_{ij,i} + \dot{f}_j = \rho \frac{\partial^2 v_j}{\partial t^2}, \tag{4}$$

where ρ is the mass density, $\dot{f}_j$ the incremental body force, and t denotes the time. Considering a time-harmonic motion with circular frequency Ω, the incremental displacement field $v_i(\mathbf{x}) \exp(-i\Omega t)$ can be derived from a stream function $\psi(\mathbf{x}) \exp(-i\Omega t)$, introduced as

$$v_1 = \psi_{,2}, \quad v_2 = -\psi_{,1}. \tag{5}$$

A substitution of equation (1) and (5) in equation (4) leads to the differential equation

$$(1+k)\,\psi_{,1111} + 2\,(2\xi - 1)\,\psi_{,1122} + (1-k)\,\psi_{,2222} + \frac{\dot{f}_{1,2}}{\mu} - \frac{\dot{f}_{2,1}}{\mu} + \\ + \frac{\rho}{\mu}\Omega^2\,(\psi_{,11} + \psi_{,22}) = 0, \tag{6}$$

where the parameters k and ξ are respectively the dimensionless deviatoric prestress and the dimensionless parameter quantifying the amount of orthotropy

$$k = \frac{\sigma}{2\mu}, \qquad \xi = \frac{\mu_*}{\mu}. \tag{7}$$

The differential equation (6), defines the regime classification in terms of the following coefficients

$$\left.\begin{array}{c}\gamma_1 \\ \gamma_2\end{array}\right\} = \frac{1 - 2\mu_*/\mu \pm \sqrt{(1 - 2\mu_*/\mu)^2 + k^2 - 1}}{1+k}. \tag{8}$$

The coefficients (8) can assume two real and negative values (in the so-called 'elliptic imaginary regime' denoted by EI) or two complex-conjugate values (in the so-called 'elliptic complex regime' denoted by EC), so that they can belong only to elliptic range, to which the present study is restricted.

The elliptic regime is constrained by the Hill condition for a tensile prestress, that excludes every incremental bifurcation [1, 8], and is expressed as (for $\mu > 0$)

$$0 < p/\mu < 2\xi, \qquad \frac{k^2 + (p/\mu)^2}{2p/\mu} < 1, \tag{9}$$

while for compressive prestress, a surface instability occurs when [1, 9]

$$4\xi - 2p/\mu = \frac{(p/\mu)^2 - 2p/\mu + k^2}{\sqrt{1-k^2}}. \tag{10}$$

By solving equation (6), considering the body force as a Dirac delta function $\dot{f}_j \delta(\mathbf{x})$, the incremental displacement $\tilde{v}_i^g$ in the transformed domain of a plane-wave expansion [2] can be found in the form

$$\begin{aligned}\tilde{v}_i^g(\boldsymbol{\omega}\cdot\mathbf{x}) = \; & \frac{(\delta_{1i}\omega_2 - \delta_{2i}\omega_1)(\delta_{1g}\omega_2 - \delta_{2g}\omega_1)}{L(\boldsymbol{\omega})}\,[\mathrm{Ci}(\eta \mid \boldsymbol{\omega}\cdot\mathbf{x}\mid)\cos(\eta\,\boldsymbol{\omega}\cdot\mathbf{x}) + \\ & +\mathrm{Si}(\eta\,\boldsymbol{\omega}\cdot\mathbf{x})\sin(\eta\,\boldsymbol{\omega}\cdot\mathbf{x}) - i\frac{\pi}{2}\cos(\eta\,\boldsymbol{\omega}\cdot\mathbf{x})],\end{aligned} \tag{11}$$

where Ci and Si are the cosine integral and sine integral functions, respectively, and

$$L(\boldsymbol{\omega}) = \mu(1+k)\omega_2^4\left(\frac{\omega_1^2}{\omega_2^2} - \gamma_1\right)\left(\frac{\omega_1^2}{\omega_2^2} - \gamma_2\right) > 0, \tag{12}$$

with

$$\eta = \Omega\sqrt{\frac{\rho}{L(\boldsymbol{\omega})}}. \tag{13}$$

The infinite-body Green's function can be expressed in a final form as

$$v_i^g(\mathbf{x}) = -\frac{1}{4\pi^2}\oint_{|\boldsymbol{\omega}=1|}\tilde{v}_i^g(\boldsymbol{\omega}\cdot\mathbf{x})d\boldsymbol{\omega}, \tag{14}$$

and its gradient as

$$v_{i,k}^g(\mathbf{x}) = -\frac{1}{4\pi^2}\oint_{|\boldsymbol{\omega}|=1}\tilde{v}_{i,k}^g(\boldsymbol{\omega}\cdot\mathbf{x})d\omega, \tag{15}$$

where

$$\tilde{v}_{i,k}^g(\boldsymbol{\omega}\cdot\mathbf{x}) = \omega_k\frac{\delta_{ig} - \omega_i\omega_g}{L(\boldsymbol{\omega})}\left[\frac{1}{\boldsymbol{\omega}\cdot\mathbf{x}} - \eta\Xi(\eta\boldsymbol{\omega}\cdot\mathbf{x})\right] \tag{16}$$

and

$$\Xi(\alpha) = \sin(\alpha)\mathrm{Ci}(|\alpha|) - \cos(\alpha)\mathrm{Si}(\alpha) - i\frac{\pi}{2}\sin(\alpha). \tag{17}$$

Finally the Green's function for incremental nominal stresses can be derived from the constitutive equations (1) as

$$\begin{aligned} \dot{t}_{11}^g &= (2\mu_* - p)\,v_{1,1}^g + \dot{\pi}^g, & \dot{t}_{12}^g &= (\mu - p)\,v_{1,2}^g + (\mu + \mu k)\,v_{2,1}^g, \\ \dot{t}_{21}^g &= (\mu - p)\,v_{2,1}^g + (\mu - \mu k)\,v_{1,2}^g, & \dot{t}_{22}^g &= -\,(2\mu_* - p)\,v_{1,1}^g + \dot{\pi}^g. \end{aligned} \tag{18}$$

2 The J-2 deformation theory of plasticity

The J_2–deformation theory of plasticity can be expressed in plane strain through the constitutive equation [10]

$$\sigma_1 - \sigma_2 = K \left(\frac{2}{\sqrt{3}} \right)^{N+1} |\varepsilon_1|^{N-1} \varepsilon_1, \tag{19}$$

where K is a stiffness parameter, $N \in (0, 1]$ a hardening exponent and $\varepsilon_1 = -\varepsilon_2$ are the logarithmic strains, related to the principal stretches $\lambda_1 = 1/\lambda_2$ via $\varepsilon_1 = \log \lambda_1 = -\varepsilon_2 = -\log \lambda_2$. The incremental moduli μ and μ_*, defining equation (1), follow as

$$\mu = \frac{1}{3} E_s \left(\varepsilon_1 - \varepsilon_2\right) \coth\left(\varepsilon_1 - \varepsilon_2\right), \qquad \mu_* = \frac{1}{9} \frac{E_s}{\varepsilon_e^2} \left[3(\varepsilon_1 + \varepsilon_2)^2 + N(\varepsilon_1 - \varepsilon_2)^2 \right], \tag{20}$$

where E_s is the secant modulus to the effective-stress/effective-strain curve, given by

$$E_s = K \left(\frac{2}{\sqrt{3}} \right)^{N-1} |\varepsilon_1|^{N-1}. \tag{21}$$

The parameters (7) for a J_2–deformation theory, can be written in the form

$$k = \frac{1}{\coth(2\varepsilon_1)}, \qquad \xi = \frac{N}{2\varepsilon_1 \coth(2\varepsilon_1)}. \tag{22}$$

3 The integral equation for a shear band

The shear band can be idealized with a discontinuity surface across which the incremental quantities can suffer finite jumps. In particular, by introducing the jump operator [[ ]] as

$$[[g]] = g^+ - g^-, \tag{23}$$

[where g^+ and g^- denote the limits approached by the field $g(\mathbf{x})$ at the discontinuity surface], the boundary conditions holding along the shear band are the following:

- Null incremental nominal shearing tractions:

$$\hat{t}_{21}(\hat{x}_1, 0^\pm) = 0, \quad \forall |\hat{x}_1| < l. \tag{24}$$

- Continuity of the incremental nominal traction orthogonal to the shear band:

$$[[\hat{t}_{22}(\hat{x}_1, 0)]] = 0, \quad \forall |\hat{x}_1| < l. \tag{25}$$

- Continuity of the incremental displacement component orthogonal to the shear band:

$$[[\hat{v}_2(\hat{x}_1, 0)]] = 0, \quad \forall |\hat{x}_1| < l, \tag{26}$$

related to the reference systems $\hat{x}_1 - \hat{x}_2$ aligned parallel to the shear band. Note that the above conditions do not correspond to a sliding surface [5].

The incremental displacement of a time-harmonic motion, with circular frequency Ω, is described by an incident plane wave with phase velocity c in a direction defined by the unit propagation vector $\mathbf{p}$ and assuming the form [11]

$$\mathbf{v}^{inc} = A\mathbf{d}e^{i\frac{\Omega}{c}(\mathbf{x}\cdot\mathbf{p}-ct)}, \tag{27}$$

where A is the amplitude and $\mathbf{d}$ is the direction of motion. Since the wave (27) propagates in an incompressible material, isochoricity implies

$$\mathbf{d}\cdot\mathbf{p} = 0, \tag{28}$$

so that the incident wave is transverse, with the motion orthogonal to the propagation direction. A substitution of equation (27) into equation (6), written with $\dot{f}_{1,2} = \dot{f}_{2,1} = 0$, and use of equation (28) yield the following expression for the wave speed

$$c^2 = \frac{\mu}{\rho}\left[(1+k)p_1^4 + 2\left(2\xi - 1\right)p_1^2 p_2^2 + (1-k)p_2^4\right], \tag{29}$$

which, setting $p_1 = \cos\beta$ and $p_2 = \sin\beta$ and

$$c_1 = \sqrt{\mu(1+k)/\rho}, \tag{30}$$

provides

$$c(\beta) = c_1 \sin^2\beta \sqrt{\left(\cot^2\beta - \gamma_1\right)\left(\cot^2\beta - \gamma_2\right)}. \tag{31}$$

A scattered incremental displacement field $\mathbf{v}^{sc}(\mathbf{x})e^{-i\Omega t}$ is generated by the interaction of the incident wave with the shear band such that the total incremental displacement field $\mathbf{v}(\mathbf{x})e^{-i\Omega t}$ is represented as the sum

$$\mathbf{v} = \mathbf{v}^{inc} + \mathbf{v}^{sc}. \tag{32}$$

The scattered field $\mathbf{v}^{sc}$ satisfies an extension of the Betti identity [2]

$$v_g^{sc}(\mathbf{y}) = \int_{\partial B} \left(\dot{t}_{ij} n_i v_j^g(\mathbf{x}, \mathbf{y}) - \dot{t}_{ij}^g(\mathbf{x}, \mathbf{y}) n_i v_j\right) dl_{\mathbf{x}}, \tag{33}$$

where ∂B represents the boundary of the shear band, which is made up of the two surfaces of the shear band with length $2l$, and external unit normals of opposite sign, so that equation (33) can be specialized for a shear band in the form

$$v_g^{sc}(\mathbf{y}) = -\int_{-l}^{l} \left([\![\dot{t}_{ij}]\!] n_i v_j^g(\hat{x}_1, \mathbf{y}) - \dot{t}_{ij}^g(\hat{x}_1, \mathbf{y}) n_i [\![v_j]\!]\right) d\hat{x}_1. \tag{34}$$

Due to the boundary conditions (24)–(25), the integral equation reduces to

$$v_g^{sc}(\mathbf{y}) = \int_{-l}^{l} \dot{t}_{ij}^{g}(\hat{x}_1, \mathbf{y}) n_i [\![v_j]\!] \, d\hat{x}_1, \tag{35}$$

which provides the incremental displacement at every point in the body as function of the jump of the incremental displacement $[\![v_j]\!]$ across the shear band.

The gradient of the incremental displacement can be derived from the integral equation (35) as

$$v_{g,k}^{sc}(\mathbf{y}) = -\int_{-l}^{l} \dot{t}_{ij,k}^{g}(\hat{x}_1, \mathbf{y}) n_i [\![v_j]\!] \, d\hat{x}_1, \tag{36}$$

so that from the constitutive equations (1) the incremental stress can be written as

$$\dot{t}_{lm}^{sc}(\mathbf{y}) = -\mathbb{K}_{lmkg} \int_{-l}^{l} \dot{t}_{ij,k}^{g}(\hat{x}_1, \mathbf{y}) n_i [\![v_j]\!] \, d\hat{x}_1 + \dot{p}(\mathbf{y}) \delta_{lm}. \tag{37}$$

In order to evaluate the unknown incremental displacement jump $[\![v_j]\!]$, the source point $\mathbf{y}$ is assumed to approach the shear band boundary. Denoting with $\mathbf{s}$ the unit vector tangent to the shear band, the boundary condition at the shear band become

$$\mathbf{n} \cdot \dot{\mathbf{t}}^{(sc)} \mathbf{s} = -\mathbf{n} \cdot \dot{\mathbf{t}}^{(inc)} \mathbf{s}, \tag{38}$$

so that equation (35) can be rewritten as

$$\hat{\dot{t}}_{21}^{(inc)}(\mathbf{y}) = n_l s_m \mathbb{K}_{lmkg} \int_{-l}^{l} \dot{t}_{ij,k}^{g}(\hat{x}_1, \mathbf{y}) n_i [\![v_j]\!] \, d\hat{x}_1. \tag{39}$$

Equation (39) represents the boundary integral formulation for the dynamics of a shear band interacting with an impinging wave. The kernel of the integral equation (39) is hypersingular of order r^{-2} as $r \to 0$, being r the distance between field point $\mathbf{x}$ and source point $\mathbf{y}$, so it is specified in the finite-part Hadamard sense.

The components of the vector of incremental displacements $\mathbf{v}$ in the reference system x_1–x_2, can be expressed in the local inclined reference system $\hat{x}_1$–$\hat{x}_2$ as

$$\mathbf{v} = \mathbf{Q} \hat{\mathbf{v}}, \quad [\mathbf{Q}] = \begin{bmatrix} \cos\vartheta & -\sin\vartheta \\ \sin\vartheta & \cos\vartheta \end{bmatrix}, \tag{40}$$

so that, due to the boundary conditions (26)

$$[\![v_j]\!] = Q_{j1} [\![\hat{v}_1]\!] = s_j [\![\hat{v}_1]\!], \tag{41}$$

equation (39) can be given the final form

$$\hat{\dot{t}}_{21}^{(inc)}(\mathbf{y}) = n_l s_m \mathbb{K}_{lmkg} \int_{-l}^{l} \dot{t}_{ij,k}^{g}(\hat{x}_1, \mathbf{y}) n_i s_j \, [\![\hat{v}_1]\!] \, d\hat{x}_1, \tag{42}$$

showing that the dynamics of a shear band is governed by a linear integral equation in the unknown jump of tangential incremental displacement across the shear band faces, $[\![\hat{v}_1]\!]$.

4 Collocation method

The integral equation (40) can be numerically solved by using a collocation technique with two different kinds of shape functions: quadratic for the elements inside the shear band and quarter-point at the tips, Figure 1(a). The quarter-point element is a quadratic element with the mid-node moved at the quarter of the lenght of the element from the tip [13], so that the shape functions describe the square root singularity present at the shear band tips, as is usual for the crack tip problem [12, 14, 15]. The quadratic shape functions are

$$\phi_1 = 1 - 3\,\zeta + 2\,\zeta^2, \tag{43}$$
$$\phi_2 = 4\,\zeta - 4\,\zeta^2, \tag{44}$$
$$\phi_3 = 2\,\zeta^2 - \zeta, \tag{45}$$

while the shape functions for the quarter point element become

$$\phi_1 = 4\sqrt{\zeta} - 4\,\zeta, \tag{46}$$
$$\phi_2 = 2\,\zeta - \sqrt{\zeta}. \tag{47}$$

Hence, using a collocation method, a system of $Q-1$ algebraic equations is obtained which can be written in matrix form as follows

$$\left\{\hat{\mathbf{t}}_{21}^{(inc)}\right\} = [\mathbf{C}]\,\{[\![\hat{\mathbf{v}}]\!]\}\,. \tag{48}$$

A validation of the numerical approach, is pursued through an analysis of a shear band present in an isotropic material at null prestress, that can be compared with a crack loaded in Mode II. Figure 1 (b) shows the results of the normalized SIF function of the wavenumber, for three different inclinations of the wave propagation vector $(0, \pi/6, \pi/3)$. The solution obtained with quadratic and quarter point elements (Q+QP, circle spots) is compared with an available analytical solution [6] (solid lines), while another reported is based on linear and square root shape functions [7] (L+SR, diamond spots). With a discretization of 100 elements the errors related to the analytical solution is about 8% for the mixed BEM with $L + SR$ and about 0.2% for the collocation technique with $Q + QP$ employed in our study.

References

[1] Bigoni, D. (2012) Nonlinear Solid Mechanics Bifurcation Theory and Material Instability. Cambridge University Press.

[2] Bigoni, D. and Capuani, D., Bonetti, P. and Colli, S. (2007) A novel boundary element approach to time-harmonic dynamics of incremental nonlinear elasticity: The role of pre-stress on structural vibrations and dynamic shear banding. Comput. Meth. Appl. Mech. Engrg. 196, 4222-4249.

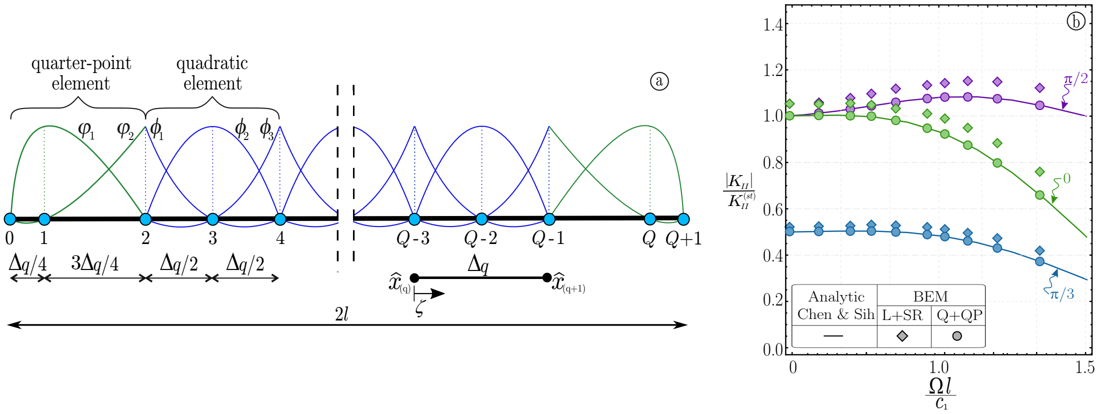

Figure 1: (a) Subdivision of the shear band line in Q-elements. Within each elements a quadratic variation of the incremental displacement jump is assumed, with the exception of the two elements at the shear band tips where the incremental displacement jump assume a quarter point variation. (b) Comparison of the modulus of the normalized mode II Stress Intensity Factor at the shear band tip (for an isotropic material at null prestress) as a function of the wavenumber, with the analytical solution of Chen and Sih[6] and the numerical solution of Giarola[7].

[3] Bigoni, D., Dal Corso, F. (2008) The unrestrainable growth of a shear band in a prestressed material. Proc. R. Soc. A 464, 2365-2390.

[4] Biot, M.A. (1965) *Mechanics of incremental deformations*. J. Wiley and Sons, New York.

[5] Bigoni, D., Bordignon, N., Piccolroaz, A., Stupkiewicz, S. (2018) Bifurcation of elastic solids with sliding interfaces. Proc. R. Soc. A 474 20170681.

[6] Chen, E.P., Sih, G.C. (1977) Scattering waves about stationary and moving cracks. *Mechanics of fracture:Elastodynamic crack problems*, pp. 119-212. Noordhoff, Leyden.

[7] Giarola, D., Capuani, D., Bigoni, D. (2017) The dynamics of a shear band. J. Mech. Phys. Solids 112, 472-490.

[8] Hill, R. (1958) A general theory of uniqueness and stability in elastic-plastic solids. J. Mech. Phys. Solids 6, 236-249.

[9] Hill, R., Hutchinson, J.W. (1975) Bifurcation phenomena in the plane tension test. J. Mech. Phys. Solids 23, 239-264.

[10] Hutchinson, J.W., Neale, K.W. (1979) Finite strain J2-deformation theory. In Proc. IUTAM Symp. on Finite Elasticity (eds D. E. Carlson and R. T. Shield), pp. 237-247. The Hague, The Netherlands: Martinus Nijhoff.

[11] Ogden, R., Singh, B. (2011) Propagation of waves in an incompressible transversely isotropic elastic solid with initial stress: Biot revisited. J. Mech. Materials Struct. 6, 453-477.

[12] Paulino, G.H., Gray, L.J. (1998) Crack Tip Interpolation, Revisited. SIAM Journal on Applied Mathematics. 58, 428-455.

[13] Salvadori, A. (2002) Analytical integrations in 2D BEM elasticity. Comput. Meth. Appl. Mech. Engrg. 53, 1695-1719.

[14] Salvadori, A. , Gray, L.J. (2007) Analytical integrations and SIFs computation in 2D fracture mechanics. Comput. Meth. Appl. Mech. Engrg. 70, 445-495.

[15] Tan, A., Hirose, S., Zhang, Ch. (2005) A time-domain collocation-Galerkin BEM for transient dynamic crack analysis in anisotropic solids. Engineering Analysis with Boundary Elements 29, 1025-1038.
